# Direct Ink Writing of Three-Dimensional (K, Na)NbO_3_-Based Piezoelectric Ceramics

**DOI:** 10.3390/ma8041729

**Published:** 2015-04-14

**Authors:** Yayun Li, Longtu Li, Bo Li

**Affiliations:** 1 State Key Laboratory of New Ceramics and Fine Processing, School of Materials Science and Engineering, Tsinghua University, Beijing 100084, China; E-Mail: llt@mail.tsinghua.edu.cn; 2 Research Institute for Advanced Materials, Graduate School at Shenzhen, Tsinghua University, Shenzhen 518055, China; E-Mail: Boli@mail.tsinghua.edu.cn

**Keywords:** (K, Na)NbO_3_ (KNN), direct ink writing, 3D structures

## Abstract

A kind of piezoelectric ink was prepared with Li, Ta, Sb co-doped (K, Na)NbO_3_ (KNN) powders. Piezoelectric scaffolds with diameters at micrometer scale were constructed from this ink by using direct ink writing method. According to the micro-morphology and density test, the samples sintered at 1100 °C for 2 h have formed ceramics completely with a high relative density of 98%. X-ray diffraction (XRD) test shows that the main phase of sintered samples is orthogonal (Na_0.52_K_0.4425_Li_0.0375_)(Nb_0.87_Sb_0.07_Ta_0.06_)O_3_. The piezoelectric constant d_33_ of 280 pC/N, dielectric constant ε of 1775, remanent polarization P_r_ of 18.8 μC/cm^2^ and coercive field E_c_ of 8.5 kV/cm prove that the sintered samples exhibit good electrical properties. The direct ink writing method allows one to design and rapidly fabricate piezoelectric structures in complex three-dimensional (3D) shapes without the need for any dies or lithographic masks, which will simplify the process of material preparation and offer new ideas for the design and application of piezoelectric devices.

## 1. Introduction

Environmentally-friendly lead-free piezoelectric ceramics have attracted much attention in recent years. In 2004, Saito *et al.* [1] reported a big breakthrough of a high d_33_ (416 pC/N) in the Li^+^-, Ta^5+^-, and Sb^5+^-modified (K, Na)NbO_3_ (KNN) textured ceramics as a promising candidate for lead-free piezoelectric ceramics. Up to now, studies about KNN are focusing on the improvement of the materials and the structure designs. Material improvement mainly concerns the substitution behavior [2,3], phase transitional behavior [4,5,6,7] and adulteration of the materials [8,9,10,11,12]. For the structure design refers to the KNN-based piezoelectric devices such as the sensors, transducers and actuators [13,14,15,16,17], most of which are fabricated by the 1–3 KNN composite [18,19,20] or the single crystalline KNN nanostructures [21,22,23,24]. Few works about the three-dimensional (3D) KNN structures, however, have been reported in the fabrication of piezoelectric devices. Direct ink writing (DIW) technique is a powerful fabrication method in building 3D KNN piezoelectric scaffolds due to its favorable performance. In this method, subtle 3D KNN architectures can be constructed without the need for any dies or lithographic masks [25], which is more convenient than the dice and fill method to fabricate 1–3 type KNN products [26]. DIW shows a potential for application in catalytic materials [27], tissue engineering [28] and especially the composites [29].

In this paper, we prepared methyl methacrylate based suspensions of KNN inks and fabricated 3D piezoelectric woodpile structures with various structural parameters by using DIW technique. The phase composition, the microstructure and the piezoelectric parameters of the sintered samples were observed. 

## 2. Experimental Section

The KNN-based powders with enhanced piezoelectricity by co-additive of Li, Sb, and Ta were prepared by a two-step calcining and ball milling route. The average particle size of the powder was about 0.5 μm with the specific surface area of 10.1 m^2^/g. Methyl methacrylate (AR, Beijing Yili Fine Chemicals, Beijing, China) and pentaerythritol three acrylate (Alfa Aesar, Beijing, China) were mixed as a solvent. A certain amount of KNN powders were added into the mixture with ultrasound treatment and the final suspension had a solid content fraction of 56 wt%. The plot of viscosity as a function of the shear stress and the plot of shear stress on shear rate were measured by a controlled stress rheometer (Physica MRC 300 Modular Compact Rheometer, Physica Paar, Berlin, Germany) under the pressure from 1 Pa to 1000 Pa at room temperature.

**Figure 1:**
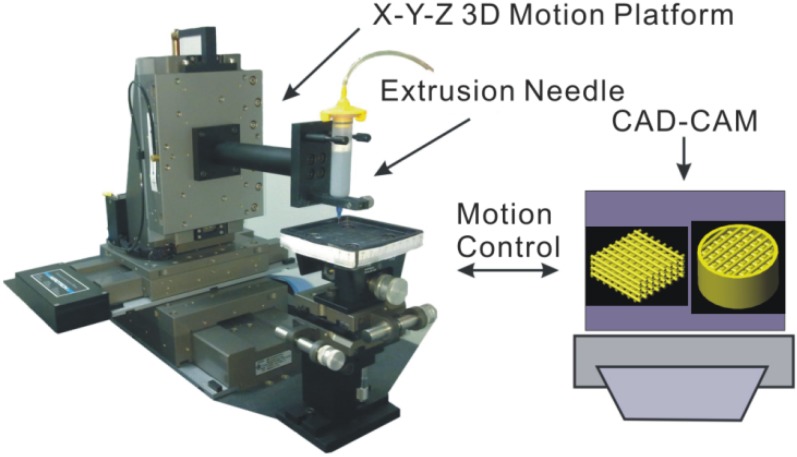
Experimental set-up for the direct write printing process.

The direct ink writing equipment (A3200, Aerotech Inc., Pittshurgh, PA, USA) consists of three parts (Figure 1). The first one is the computer-controlled system which designs the structure and translates the mobile paths into code languages. The second one is the 3D motion platform which can move under the instruction of the computer. The last one is the extrusion system which can eject the inks under a proper pressure. In this experiment, we designed three woodpile structures with different sizes and changed those into code languages. The 3D platform constructed all the KNN woodpile structures after receiving the motion instructions.

Those green samples were sintered at varying temperatures for 2 h with a heating rate of 3 °C/min. The macrostructures of the samples were characterized by optical microscope (AXio Imager.Z1m, Carl Zeiss Shanghai Co. Ltd., Shanghai, China). The microstructures of the sintered samples were analyzed by using scanning electron microscopy (Leo-1530, Oberkochen, Germany). The density of the sintered sample was measured by the Archimedes technique with deionized water as the suspension fluid. The dielectric properties were measured by an impedance analyzer (HP4278A, Santa Clara, CA, USA) and the d_33_ measurements were performed by using a ZJ-3A quasi-static d_33_ tester (Institute of Acoustics, Chinese Academy of Sciences, Beijing, China). The hysteresis loop was tested by a ferroelectric tester (RT 6000HVA, Radiant Technologies Inc., Albuquerque, NM, USA) under a maximum electric field of 2.5 kV/mm.

## 3. Results and Discussion

**Figure 2:**
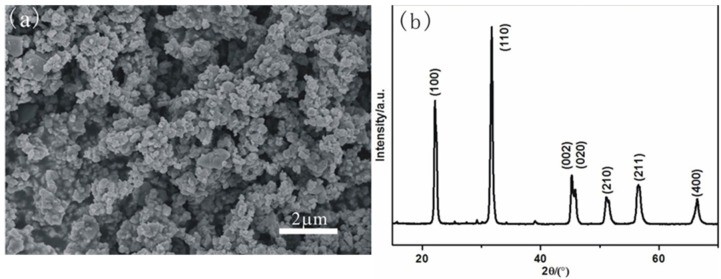
(**a**)The SEM image of the KNN powders; (**b**) the XRD pattern of the KNN powders.

Nanosize KNN powders with little soft-agglomerates is shown in Figure 2a. The X-ray diffraction (XRD) pattern of the KNN powders (Figure 2b) shows the main phase is (Na_0.52_K_0.4425_Li_0.0375_)(Nb_0.87_Sb_0.07_Ta_0.06_)O_3_ which is prepared by the co-additive of Li, Sb, and Ta in the (K, Na)NbO_3_ powders.

**Figure 3:**
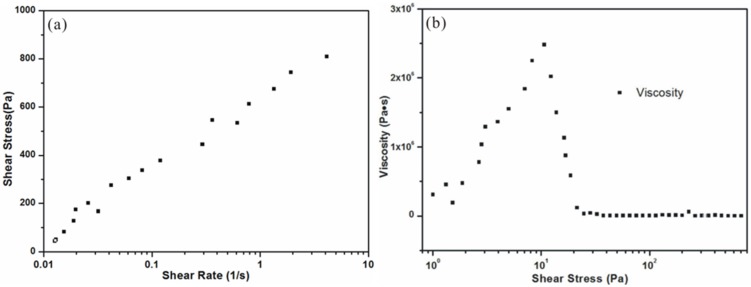
(**a**) Plot of the shear stress as a function of shear rate; (**b**) plot of the viscosity as a function of shear stress.

Figure 3a shows the plot of shear stress as a function on shear rate, which is close to that of the classic shear thinning fluid. When the shear stress less than 15 Pa, the ink shows an elastic deformation behavior, and the deformation can be restored after unloading (Figure 3b). Then the viscosity decreases rapidly and experiences a plastic deformation to reach a steady state at 50 Pa. This deformation type cannot be restored after unloading. The changing trend of the plot indicates that the ink has a shear-thinning behavior.

The optimum solid content of the ink is related to the specific surface area of the original powders, and the KNN ink with a solid content fraction of 56 wt% is beneficial for the fabrication process and can be sintered with a high relative density. For example, the ink formed by TiO_2_ nanoparticle powders (specific surface area of 50 m^2^/g) has a proper solid content of 42.5 wt% [30]. The ink formed by PLZT powders (specific surface area of 4.735 m^2^/g) has an appropriate solid content about 70 wt% [31]. Both of the above mentioned two inks can be sintered with a high relative density. In this paper, we use the KNN powders with a specific surface area of 10.1 m^2^/g to form the ink with a final solid content of 56 wt%. We can conclude that the powders with a larger specific surface area can be developed to an ink with a smaller solid content fraction. 

**Figure 4:**
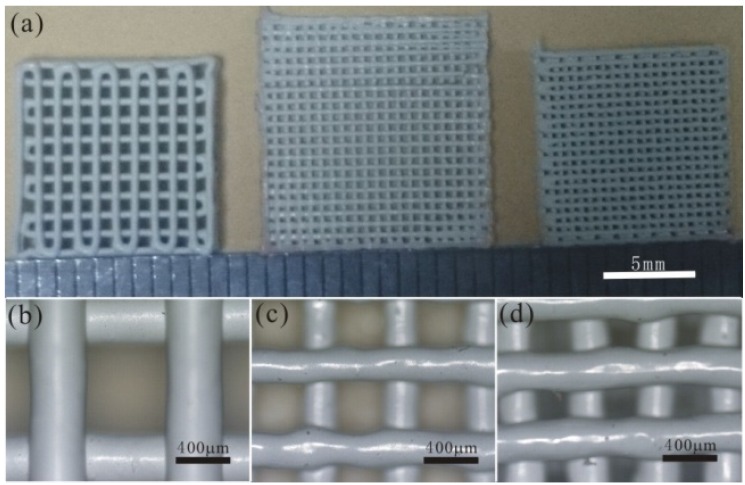
Optical images of the woodpile structures: (**a**) top view of three samples with different sizes; (**b**) 10 mm × 10 mm sample with a filament diameter of 400 μm; (**c**) 12 mm × 12 mm sample with a filament diameter of 250 μm; (**d**) 10 mm × 10 mm sample with filament a diameter of 300 μm.

Here, we design 3D KNN woodpile structures with various shape sizes, filament diameters and rod-to-rod distances. Figure 4a shows the top view of the three structures with shape sizes of 10 mm × 10 mm or 12 mm × 12 mm. Figure 4b shows the sample with the filament diameter of 400 μm and rod-to-rod distance of 1 mm. Figure 4c shows the sample with the filament diameter of 250 μm and rod-to-rod distance of 600 μm. Figure 4d shows the sample with the filament diameter of 300 μm and rod-to-rod distance of 500 μm. It is obvious that the direct ink writing (DIW) method has an advantage in the structure design and size precision control.

**Figure 5:**
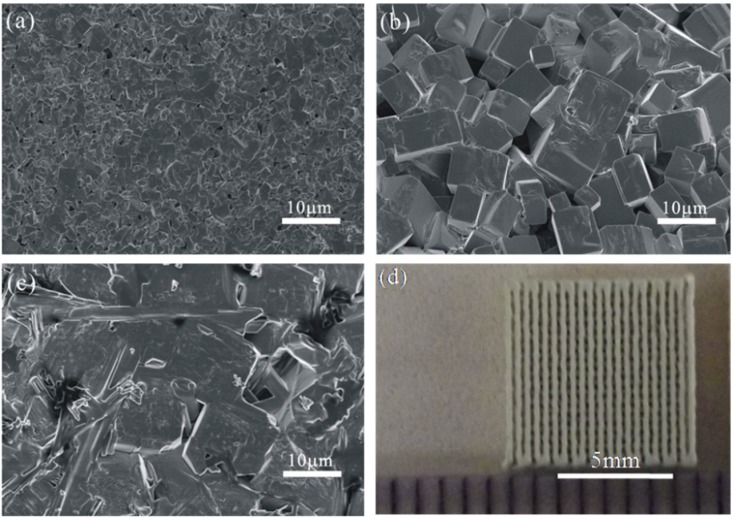
SEM photos of KNN samples sintered to varying maximum temperatures: (**a**) 1050 °C; (**b**) 1100 °C; (**c**) 1130 °C; (**d**) the top view of the sintered 10 mm × 10 mm sample.

A series of sintered experiments have been performed to find out the optimum sintering process. We designed three sintering temperatures, namely: 1050 °C, 1100 °C and 1130 °C. Figure 5a shows the sample sintered in 1050 °C for 2 h, we can see that this sample has not been sintered completely with the non-uniform grain size and lots of pores. Figure 5c shows the SEM image of the sample sintered under 1130 °C for 2 h, which is over sintered with an abnormal grain growth. From Figure 5b we can conclude that the KNN sample sintered at 1100 °C for 2 h has a better performance with the uniform grain size and less pores. The density of the sintered sample was measured by the Archimedes method with deionized water as the suspension fluid. Moreover, the testing density is 4.52 g/cm^3^, while the theoretical density of KNN is about 4.60 g/cm^3^. The relative density (98%) is the ratio of the testing density and the theoretical density. Figure 5d shows the top view of the sample (on the right-hand side of Figure 4a) sintered at 1100 °C for 2 h, which shows that the deformation did not occur during the sintering process.

**Figure 6:**
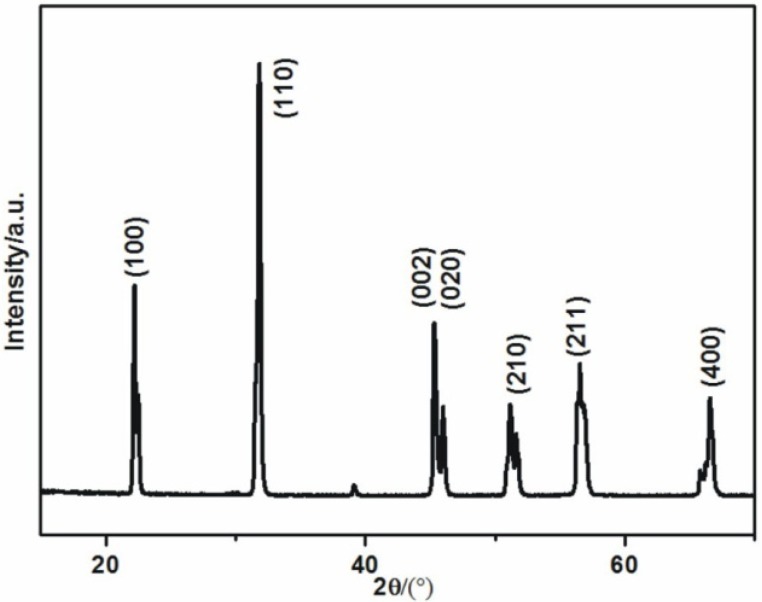
XRD pattern of the sample sintered at 1100 °C.

Figure 6 shows the XRD pattern of the KNN sample sintered at 1100 °C. The sintered sample agrees well with the origin powders, indicating that no significant phase transition occurred during the sintering process.

**Figure 7:**
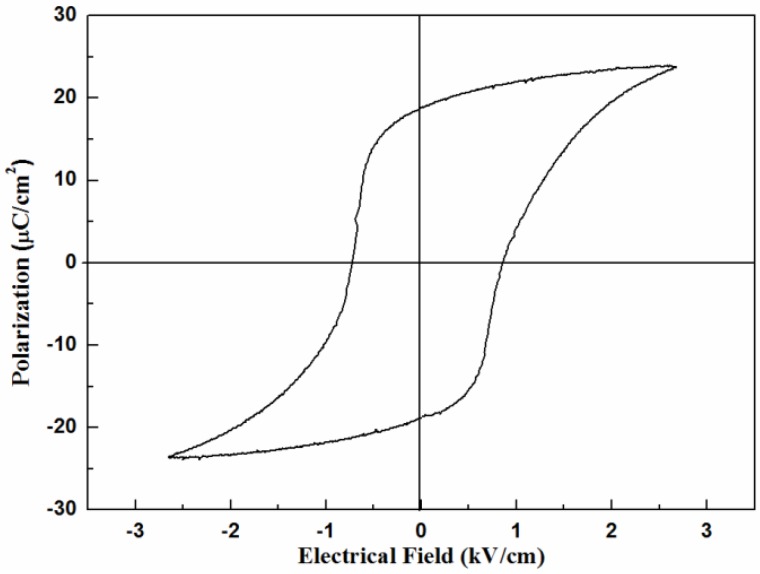
The hysteresis loop of the sintered KNN sample.

The sintered sample with the original size of 10 mm×10 mm and filament diameter of 400 μm was printed with the low temperature silver paste on the top and bottom surface and covered with aluminum foil. Then the sample was poled at 2.5 kV/mm for 20 min in a silicone oil bath at 100 °C. The hysteresis loop was tested under a maximum electric field of 2.5 kV/mm (Figure 7), and the value of remanent polarization P_r_ and coercive field E_c_ were gained. Table 1 shows the dielectric and piezoelectric properties of the sintered 3D KNN sample. We can see that sintered sample has good electrical properties with piezoelectric constant d_33_ of 280 pC/N, dielectric constant of 1775, remanent polarization P_r_ of 18.8 μC/cm^2^ and coercive field E_c_ of 8.5 kV/cm.

**Table 1:** Dielectric and piezoelectric properties of the sintered KNN sample.

Electrical properties	ε_33_/ε_0_	d_33_ (pC/N)	P_r_ (μC/cm^2^)	E_c_ (kV/cm)
KNN sample	1775	280	18.8	8.5

## 4. Conclusions

We prepared KNN woodpile structures with a relatively high piezoelectric constant by using direct ink writing method along with the ink methyl methacrylate based suspensions of KNN. Three woodpile structures with various shape sizes, filament diameters and rod-to-rod distances have been constructed. Samples sintered in 1100 °C for 2 h have a relative density of 98%. The piezoelectric constant d_33_ of 280 pC/N, dielectric constant ε of 1775, remanent polarization P_r_ of 18.8 μC/cm^2^ and coercive field E_c_ of 8.5 kV/cm show that the sintered samples exhibit good electrical properties. Compared with the other methods such as dice and fill method and injection molding, the direct ink writing method allows one to design and rapidly fabricate piezoelectric structures in complex 3D shapes without the need for any dies or lithographic masks, which will simplify the processing of the material preparation and offer ideas for the design and application of new piezoelectric devices.
